# Food Structure Modulation of Antioxidant Bioaccessibility and Gut Epithelial Protection: The Case of Bread and Pasta Made With Pigmented Wheat

**DOI:** 10.1002/fsn3.71863

**Published:** 2026-05-03

**Authors:** Giulia Feliziani, Marianna Tagliasco, Xinying Suo, Nicoletta Pellegrini, Elena Vittadini, Laura Bordoni, Rosita Gabbianelli

**Affiliations:** ^1^ School of Advanced Studies University of Camerino Camerino Italy; ^2^ Unit of Molecular Biology and Nutrigenomics University of Camerino Camerino Italy; ^3^ Department of Agricultural, Food, Environmental and Animal Sciences University of Udine Udine Italy; ^4^ School of Biosciences and Veterinary Medicine University of Camerino Camerino (MC) Italy

**Keywords:** antioxidant activity, Caco‐2 cells, food matrix, in vitro digestion, pigmented wheat, starch digestibility

## Abstract

Food structure is expected to play an important role in modulating nutrient bioavailability and antioxidant activity in cereal‐based foods. This study examined how semolina type [pigmented grains (Grano Mischio, GM) vs. traditional wheat (Senatore Cappelli, SC)] and the food matrix (bread vs. pasta) influence product quality, in vitro starch digestibility, antioxidant capacity, and intestinal barrier integrity of Caco‐2 cells. GM‐bread had lower specific volume and was harder than SC‐bread, while GM‐pasta showed significantly higher starch digestibility compared to SC‐pasta, probably due to polyphenol‐induced structure weakening. Antioxidant activity, measured by ORAC, DPPH, and ABTS assays, varied by matrix and method, with GM‐pasta exhibiting higher radical scavenging capacity than GM‐bread in the DPPH and ABTS assays. Caco‐2 cells treated with digested GM‐pasta showed increased viability, enhanced transepithelial electrical resistance, and reduced inflammatory markers (IL‐1β, IL‐11, NF‐κB) under pro‐inflammatory conditions. Overall, pigmented wheat products, particularly pasta, retain antioxidant properties upon digestion. These findings provide evidence that food processing can modulate the biological properties of raw cereal materials, laying a promising foundation for the rational design of functional foods that leverage matrix architecture to optimize the release and efficacy of bioactive compounds during digestion.

## Graphical abstract

Pigmented wheat was processed into pasta and bread and subjected to in vitro digestion. The resulting bioaccessible fractions were characterized for antioxidant capacity and used to treat Caco‐2 epithelial monolayers under inflammatory conditions. Compared with control wheat, pigmented pasta retained higher antioxidant activity and improved intestinal barrier integrity, reducing NF‐κB expression. This image was created in BioRender. To access the original source of this presentation, visit: https://app.biorender.com/illustrations/690ca6b59c9bb31341db4209.

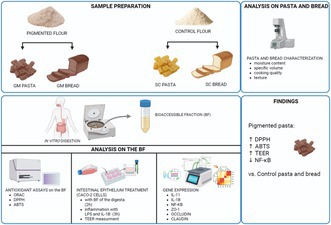

## 1 Introduction

By 2020, wheat had become the most widely cultivated crop in the world, with a global area of 220 million hectares and a production exceeding 765 million tons. It is the second most consumed cereal (as food) after rice (81 kg/year per person, 46% of total cereal consumption) and contributes 18% of total dietary calories and 19% of protein intake (Gupta et al. 2021; Reynolds and Braun 2022).

Wheat is widely consumed due to the diversity of its grain species, which are suitable for producing a large number of staple foods. *Triticum aestivum* is commonly used to make bread, pita, crackers, and snacks, while *Triticum durum* is primarily used for pasta and couscous (Reynolds and Braun 2022). In recent decades, among these two species, specific cultivars (e.g., pigmented wheat) have gained increasing popularity worldwide. This trend is driven by a desire to rediscover traditional foods that have remained largely unchanged over centuries, as well as by the richness of these grains in bioactive compounds like polyphenols and anthocyanins (Dinu et al. 2018) which have been associated with antioxidant, anti‐inflammatory, and potentially chronic disease‐preventive effects (Saini et al. 2023; Ma et al. 2020).

Different cereal products, such as pasta and bread, even when made from the same raw material, undergo distinct processing (e.g., extrusion and drying for pasta; fermentation and baking for bread), which result in products with significantly different structures (Sissons 2008). These structural differences can affect oral processing, leading to boluses of differing sizes and shapes (Suo et al. 2021), and ultimately influence the release and absorption of nutrients and bioactives during digestion (Pentikäinen et al. 2019; Tagliasco, Capuano, et al. 2025). Moreover, as shown by Vanhatalo et al. (Vanhatalo et al. 2022), denser matrices like pasta disintegrate less during digestion compared to more porous ones like bread, affecting starch digestibility and nutrient availability (Vanhatalo et al. 2022). The presence of fiber and phenolic compounds may further modify matrix formation by interfering with gluten development, thus influencing food breakdown and nutrient absorption (Lin et al. 2020; Tagliasco, Font, et al. 2024). Inclusion of wholegrain flour from pigmented grains in product formulation is expected to have positive effect in food products by boosting their antioxidant activity and delivering new sensory experiences to the consumers (Suo et al. 2023; Ed Nignpense et al. 2024). All these considerations underscore that both the intrinsic properties of the raw material and the processing methods used, by shaping the structure of product matrices, play a decisive role in modulating biological effects of food, influencing how bioactive compounds are released, transformed, and ultimately delivered during digestion. Understanding this structure–function relationship is therefore critical for the development of cereal‐based foods that can reliably provide meaningful health benefits (Martins‐Gomes et al. 2024).

However, despite promising in vitro and animal studies, evidence on the bioactivity and health effects of bioactive compounds from pigmented cereals in foods remains limited. Most existing studies have investigated purified polyphenols, including those derived from pigmented grains, using extracts rather than whole‐food matrices (Ed Nignpense et al. 2024; Francavilla and Joye 2020; Veiga et al. 2020). While these approaches are useful for identifying specific bioactive molecules, they do not account for the complex interactions occurring within real food systems, where processing, matrix structure, and interactions with proteins, starch, and dietary fiber can strongly influence the stability, bioaccessibility, and biological activity of these compounds during digestion (Shahidi and Pan 2022; Jiang et al. 2024).

Building on previous findings, the present study investigates how the higher polyphenol content of Grano Mischio (GM) (Suo et al. 2023) a mixture of pigmented wheat varieties containing both *Triticum durum* and *Triticum aestivum* species, influences the technological and structural properties of its derived products (bread and pasta), and how, in turn, food processing modulates their biological functionality, in comparison with products made with a non‐pigmented wheat variety (Senatore Cappelli, SC) (Giacosa et al. 2022). Specifically, it was evaluated whether GM food matrices (bread and pasta) retain higher antioxidant capacity following in vitro digestion and whether their digesta exert greater protective effects on intestinal epithelial integrity using the Caco‐2 cell line model than SC counterparts. This approach provides insights into how both a polyphenol‐rich raw material and the processing of cereal foods jointly contribute to shaping their nutritional and functional properties.

## 2 Materials and Methods

### 2.1 Materials

Pasta (maccheroni pugliesi‐shaped) produced using GM and SC was acquired from Pastificio Marella (Pastificio Marella S.r.l., Gioia del Colle, Bari, Italy). Pastas were produced using bronze extrusion (trafilatura al bronzo) and dried at low temperature (< 38°C for 48–72 h). GM wholemeal flour was kindly provided by CREO BIO (Gravina di Puglia, Italy), and non‐pigmented (SC) wheat semolina was provided by Pastificio Marella (Pastificio Marella S.r.l., Gioia del Colle, Bari, Italy). GM flour and SC semolina were used for bread production, along with baker's yeast and salt purchased from a local supermarket. Enzymes used to mimic gastrointestinal digestion included pepsin P7000 (from porcine gastric mucosa), pancreatin P7545 (from porcine pancreas, 8 × USP), invertase I4504 (from baker's yeast), and amyloglucosidase A7095 (from *Aspergillus niger*) (Sigma Aldrich, Milan, Italy). The total starch assay kit was bought from Megazyme International, (Ireland). For antioxidant assays, the following reagents from Sigma‐Aldrich were used: DPPH (2,2‐diphenyl‐1‐picrylhydrazyl), ABTS (2,2′‐azino‐bis‐ethylbenzothiazoline‐6‐sulfonic acid), Trolox (6‐hydroxy‐2,5,7,8‐tetramethylchroman‐2‐carboxylic acid), and potassium persulfate. For gene expression analysis, RNA was reverse transcribed using the iScript cDNA Synthesis Kit (Bio‐Rad, Italy), and qPCR was performed using SsoAdvanced Universal SYBR Green Supermix (Bio‐Rad, Italy). Primers were custom‐synthesized by Eurofins (sequences reported in Table S1). All chemicals and solvents were of analytical grade.

## 3 Methods

### 3.1 Bread Preparation

Bread was prepared using GM flour and SC semolina, following the recipe outlined in Table 1. The amount of water to add and the mixing time for each flour (GM and SC) were optimized using farinograph analysis (30°C, 63 rpm; Promylograph T6, Max Egger, Austria) to achieve a dough consistency of 500 Farinograph Units (FU). The production process was carried out following Tagliasco et al. (Tagliasco, Peressini, and Pellegrini 2025), with some modifications. After the mixing step, the dough was left to rise in an incubator (30°C, 85% humidity) for 52 min. Dough was then punctured and divided into 75 g portions that were placed on an oiled baking tray and allowed to further ferment under the same conditions. Leavened dough was then baked in a professional oven (A0S101ETA1, 17.5 kW, 400 V, 50/60 Hz, Electrolux, Stockholm, Sweden) for 30 min at 160°C, with humidity ramp decreasing from 80% to 30%. Bread loaves were cooled for 1 h at 21°C, packaged in Ziploc quart freezer bags (17.7 × 18.8 cm, SC Johnson, USA) and analyzed within 24 h. All bread productions were performed in triplicate.

**TABLE 1:** Formulation of bread (B) made with Senatore Cappelli flour (SC) and Grano Mischio (GM). The ingredients are expressed as a percentage of 100% flour.

	SC‐B	GM‐B
**Flour, g**	100	100
**Water, mL**	61	70
**Salt, g**	1	1
**Yeast, g**	1.2	1.2
**Mixing time, min**	6	6

#### 3.1.1 Pasta Cooking

Dry pasta (P) was cooked in boiling deionized water at a pasta‐to‐water ratio of 1:10 for a 14 min cooking time that was validated to be optimal (OCT). OCT was determined as reported by Suo et al. (Suo et al. 2021). Briefly, six people independently evaluated pasta samples cooked for increasing times (from 10 to 16 min at 1‐min intervals) and evaluated the products' texture (hardness and adhesiveness), determining the OCT as the moment when the pasta was perceived as cooked. Cooking water and cooked pasta were immediately separated, cooked pasta was placed into a sealed container to prevent moisture loss, and they were allowed to cool to room temperature prior to technological characterizations. Cooked pasta was not washed after cooking to reproduce typical consumption conditions. Pasta was cooked for each sample in triplicate.

#### 3.1.2 Bread Characterization

Moisture Content of the Bread Crumbs Was Determined Following the AACC Approved Method 44–15.07 (Method AACC 1999). The Specific Volume Was Calculated by Dividing the Volume of the Bread, Measured Using the Rapeseed Displacement Method (AACC Approved Method 10–05.01 (Method AACC 2009)), by Its Weight (g), with the Result Expressed in cm^3^/g. Texture Profile Analysis (TPA) Was Used to Evaluate the Textural Characteristics of the Bread Crumb. Bread Loaves Were Sliced (25 Mm Thick), and the Central Portion of the Slice Was Portioned Using a Cylindrical Mold (20 Mm Diameter). The Resulting Breadcrumb Cylinder Underwent Double Compression With a Cylindrical Probe (P/36) (36 Mm Diameter) Using a T.A. XT Plus Analyzer (Stable Micro Systems, Godalming, UK) Equipped With a 30 Kg Load Cell. The Test Parameters Were Set as Follows: 1 Mm/s Speed, 40% Compression, and a Resting Time of 5 s Between the First and Second Compression. The Textural Properties Were Obtained From the Curve Generated During the Double Compression: Hardness (Highest Force During the First Compression, N), springiness (Calculated as the Ratio of the Distance of the Second Compression to That of the First Compression, Representing the Ability of the Sample to Regain Its Original Shape After the First Compression, Dimensionless), and Cohesiveness (Calculated as the Ratio Between the Area of the Second Peak and That of the First Peak, Representing the Extent to Which the Bread Deforms When Compressed, Dimensionless) (Tagliasco, Boukid, et al. 2024). Eight to 10 Replicates Were Carried Out for Each Measurement.

#### 3.1.3 Pasta Characterization

Cooking quality: pasta solid loss was determined following the AACC Approved Method 66–50.01 (Method 66–50.01, 1989) and was expressed as g solids per 100 g of uncooked pasta (%). Cooked pasta was weighed and used to calculate the weight gain of pasta during cooking, expressed as g per 100 g uncooked pasta (%). Moisture content, texture (hardness and stickiness), and color of cooked pasta were measured following a previous report (Suo et al. 2021). Briefly, cooked pasta was dried (105°C to a constant weight), and the moisture loss represented the water content of the cooked pasta, expressed as g H_2_O per 100 g cooked pasta (%). Measurements were performed in triplicate. Pasta texture was measured using a Texture Analyzer (TA1 Texture Analyzer, AMETEK, USA) equipped with a 100 N load cell. Hardness (N) was measured using a single cutting test (cutting speed: 2 mm/s) with a flat blade (stainless steel blade: 6.4 × 11 × 0.1 cm, AMETEK, USA) and represents the peak force required for cutting the pasta strands. Stickiness (J) was evaluated by compressing the sample at 1 mm/s using an aluminum cylinder probe (36 mm × 100 mm, AMETEK, USA) at a force of 50 N (holding for 10 s) and was recorded as the work to separate the sample from the probe. At least ten measurements were analyzed for each parameter of each sample from each cooking batch. Pasta color was determined using a colorimeter (Minolta, Chroma Meter CR‐400, Japan), in terms of L* (lightness, 0: black; 100: white), a* (−: greenness; +: redness), and b* (−: blueness; +: yellowness) on cooked pasta for the OCT.

#### 3.1.4 In Vitro Digestion and Starch Digestibility of Bread and Pasta

Since starch is the main macronutrient in pasta and bread, the in vitro digestion was performed following the methodology developed by Englyst et al. (Englyst et al. 2018) and the protocol described in Tagliasco et al. (Tagliasco, Font, et al. 2024). Bread and pasta were minced using a meat mincer (Trita Express, R.G.V., Cermenate, Italy) fitted with a 7‐mm hole plate, and approximately 2 g of minced samples were digested. The sampled digesta in the intestinal phase at 20‐ and 120‐min were analyzed using the YSI 2500 Biochemistry Analyzer (Yellow Springs Instrument Company, Chicago, USA) to measure the amount of glucose released. The glucose value was converted into the amount of starch by multiplying it by 0.9. The starch released in the first 20 min of intestinal digestion was referred to as rapid digestible starch (RDS), while the starch digested between 20 min and 120 min of intestinal digestion was referred to as slowly digestible starch (SDS). The difference between the starch digested at the end of the intestinal phase and the total starch content was defined as resistant starch (RS). RDS, SDS, and RS were expressed relative to the total starch content measured in each sample. Total starch was measured using a spectrophotometric assay kit provided by Megazyme (Bray, Ireland), following the procedure for determining the total starch content in samples containing resistant starch (RTS‐NaOH Procedure—Recommended). At the end of the digestion, after sampling at 20 min and 120 min, the digesta was frozen at −20°C and then lyophilized for further analysis.

#### 3.1.5 Antioxidant Capacity of Digesta

The antioxidant activity of the digesta was assessed through ORAC, DPPH, and ABTS assays. Results were expressed as mg Trolox Equivalents (TE) per 100 g of dry weight (DW) of digesta. The ORAC assay was conducted as described by Cao et al. (Cao et al. 1993). Fluorescein (0.08 μM in 75 mM phosphate buffer, pH 7.0) was used as the fluorescent probe, and reactions were carried out in black 96‐well plates at 37°C. Trolox (6.25–50 μM) was used as the standard. Fluorescence was measured kinetically (excitation 485 nm, emission 530 nm) over 90 min, and results were expressed as TE calculated from the area under the curve (AUC), corrected for the dilution factor (1:100 v/v). The DPPH assay was carried out according to the method described by Molyneux (Molyneux 2004). In brief, a 3 mM DPPH solution in methanol was diluted to yield a working solution with an absorbance of 0.7–0.9 at 517 nm. In a 96‐well plate, 25 μL of each digested sample (diluted 1:20 v/v) was mixed with 175 μL of DPPH solution. After 15 min incubation in the dark at room temperature, absorbance was read at 517 nm. Trolox (50–150 μM) served as a standard, and antioxidant capacity was expressed as TE, corrected by the dilution factor. The ABTS assay was based on the method by Re et al. (Re et al. 1999). ABTS radical cation (ABTS^+^) was generated by reacting 7 mM ABTS with 2.45 mM potassium persulfate and incubating the solution overnight in the dark. The working solution was prepared diluting the ABTS radical solution 1:40 in ethanol to an absorbance of 0.7 ± 0.1 at 734 nm. In 96‐well plates, 25 μL of each sample (diluted 1:50 v/v) was mixed with 175 μL of ABTS solution. After 5 min of reaction, absorbance was measured at 734 nm. Trolox (25–100 μM) was used as standard. Antioxidant capacity was expressed as TE, adjusted for dilution.

#### 3.1.6 Cell Culture

To evaluate the effects of the digested food on intestinal cells, Caco‐2 cells, a human colonic epithelial cell line (ATCC, Rockville, MD, USA), were utilized. Caco‐2 cells were cultured in Dulbecco's Modified Eagle's Medium (DMEM) supplemented with 10% heat‐inactivated fetal bovine serum (FBS), 1% L‐glutamine, 1% non‐essential amino acids (NEAAs), and 1% penicillin/streptomycin. The cultures were maintained at 37°C in a humidified incubator containing 5% CO_2_. The medium was replaced every 2 days, and cells were subcultured when they reached approximately 80% confluence.

Caco‐2 cells were then grown on transwell inserts (0.4 μm pore size, ThinCert, Greiner Bio‐one, Germany) following a transwell‐based system, as outlined by Kim et al. (Kim et al. 2021). In brief, cells were seeded at a density of 26.23 × 10^3^ cells/insert in 12‐well plates, with culture medium added to both the apical (AP) and basolateral (BL) compartments. The medium was refreshed every other day, and plates were maintained at 37°C with 5% CO_2_. Caco‐2 cells were cultured until they formed a differentiated epithelial monolayer. Transepithelial electrical resistance (TEER) was measured using a Millicell ERS system (Millipore, Merck, Darmstadt, Germany) at regular intervals until the values reached a plateau, which occurred at 17 days post‐seeding.

The optimal concentration for treating cells with the digested samples was determined using the MTT assay (3‐(4,5‐dimethylthiazol‐2‐yl)‐2,5‐diphenyltetrazolium bromide) (Bahuguna et al. 2017). Cell viability was assessed at four dilutions (1:2, 1:10, 1:20, and 1:30 v/v). The highest non‐cytotoxic concentration (1:10 v/v) was selected for subsequent experiments. The same dilution factor was applied to all samples to ensure comparability across treatments as previously described in Bordoni et al. (Bordoni et al. 2024).

#### 3.1.7 Intestinal Epithelium Treatment

The intestinal epithelium was treated with the highest non‐cytotoxic concentration resulting from the MTT assay of the digesta for 2 h, simulating the time food spends in the gastrointestinal tract post‐meal (Leng et al. 1977; Boland 2016). Then, to evaluate the protective effects of the digested samples, inflammation was induced using lipopolysaccharide (LPS) and IL‐1β for 3 h (Kim et al. 2021). Specifically, on the apical compartment 0.5 mL of DMEM with LPS (10 mg/mL) was added, while on the basolateral compartment, 1.5 mL of DMEM with both LPS (10 mg/mL) and IL‐1B (10 ng/mL) was added. The treatments were performed in biological triplicates. TEER measurements were taken before and after each treatment to assess changes in epithelial barrier integrity. After the treatments, Caco‐2 cells were detached from the inserts and the resulting pellet was collected.

#### 3.1.8 Gene Expression Analysis

Total RNA was extracted from the Caco‐2 pellet using the Total RNA Purification Plus Kit (Norgen Biotek, Thorold, ON, Canada), according to the manufacturer's instructions and quantified (NanoDrop, Thermo Fisher Scientific, Italy). RNA (1 μg) was retrotranscribed to cDNA using the PrimeScript RT‐PCR Kit (Takara Bio, Göteborg, Sweden), and quantitative real‐time PCR (Biorad CFX96) was used to perform the gene expression analysis using TB Green Premix Ex Taq (Takara Bio, Göteborg, Sweden). The amplification conditions were: 30 s at 95°C (denaturation), 5 s at 95°C (annealing), and 30 s at 60°C (extension) repeated for 40 cycles. The expression levels of the target genes were normalized relative to β‐actin using the 2^−∆∆Ct^ method. Each analysis was run in technical duplicate. An inter‐run calibrator sample was applied to adjust the results obtained from different amplification plates. The target genes (IL‐11, IL‐1β, NF‐KB, ZO‐1, OCCLUDIN, CLAUDIN‐1, β‐ACT) analyzed from Caco‐2 cells are listed in the Table S1 (supporting information). Biological triplicates for each treatment were analyzed.

#### 3.1.9 Statistical Analysis

Statistical analyses were performed using SPSS (IBM SPSS Statistics for Windows, Version 29.0, Armonk, NY, USA). A *t*‐test was used to assess significant differences between the samples made with SC flour and those made with GM for each food matrix (pasta and bread). An ANOVA with Bonferroni's correction for multiple comparisons was used to compare group means. Additionally, Dunnett's test was applied to compare each sample group directly to the control group. A *p* < 0.05 was considered significant throughout the study. Results are presented as means ± standard deviation (SD).

## 4 Results

### 4.1 Bread Characteristics

**FIGURE 1:**
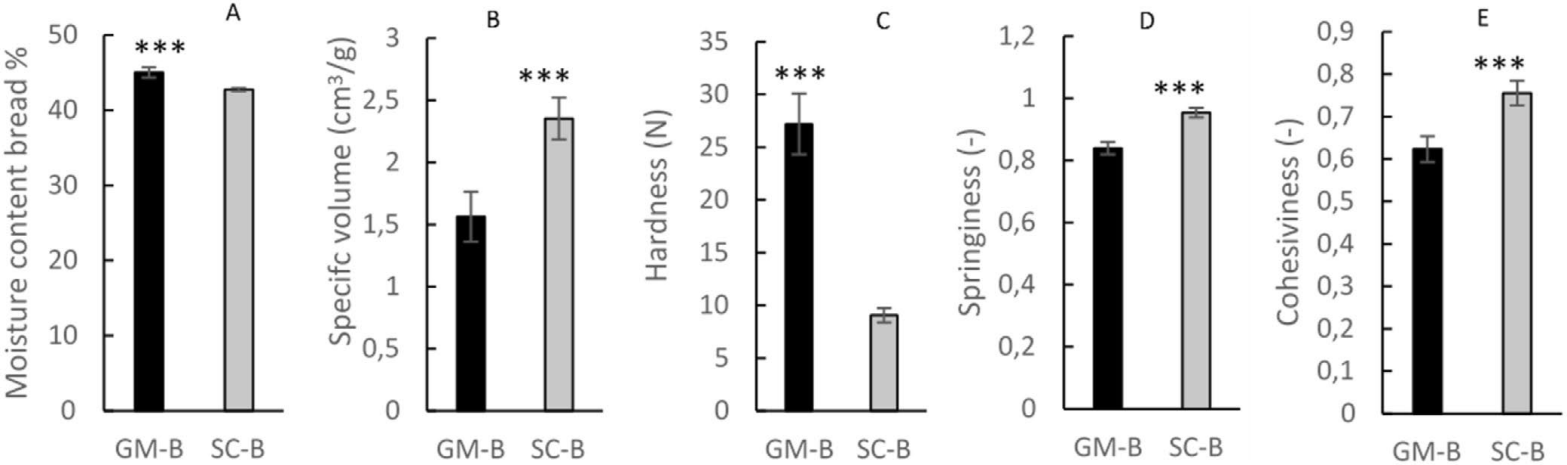
(A) moisture content (%); (B) specific volume (cm^3^/g); (C) hardness (N); (D) springiness (−); (E) cohesiveness (−) of bread made with Senatore Cappelli (SC‐B) and with Grano Mischio (GM‐B). Data are expressed as mean ± SD. *T*‐test was used to assess differences between samples: ***p < 0.001.

The characteristics of bread loaves produced with pigmented wheat (GM‐B) and common semolina (SC‐B) are shown in Figure 1. The type of flour used to produce the bread significantly affected textural features of the two loaves, even though the amount of water and the mixing time were optimized using the farinograph to achieve the same dough consistency (500°PU). SC‐B had a significantly higher specific volume (*p* < 0.001, Figure 1B) than GM‐B and was significantly softer and less wet than bread made with GM‐B (*p* < 0.001, Figure 1A,C, respectively). SC‐B was significantly springier and more cohesive than GM‐B (*p* < 0.001) (Figure 1D,E).

### 4.2 Pasta Characteristics

**FIGURE 2:**
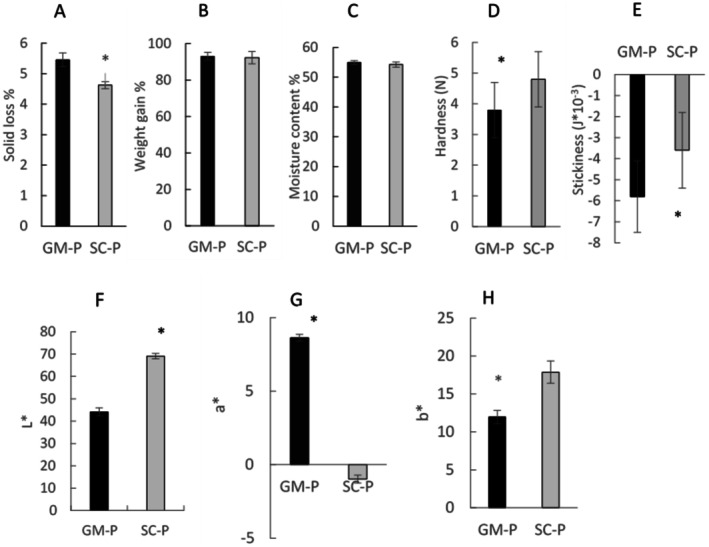
(A) Solid loss (%); (B) weight gain (%); (C) moisture content (%), (D) hardness (N), and (E) stickiness (J*10^−3^), color (F) L*: Lightness [0: Black; 100: White], (G) a* [−: Greenness; +: Redness], (H) b* [−: Blueness; +: Yellowness] of pasta made with Senatore Cappelli semolina (SC‐P) and Grano Mischio semolina (GM‐P). Data are expressed as mean ± SD. *T*‐test was used to assess the difference between samples: *p < 0.05.

Cooking quality, physical properties, and texture of cooked pastas made with pigmented (GM‐P) and non‐pigmented (SC‐P) flours are presented in Figure 2. Compared to SC‐P, GM‐P lost more solids during cooking (*p* < 0.05, Figure 2A). However, the solid loss upon cooking of both pasta samples was below 6%, indicating a good cooking quality (Morreale et al. 2019). The weight gain and moisture content of the two pasta samples do not significantly differ (Figure 2B,C). GM‐P was softer (*p* < 0.05, Figure 2D) and more adhesive (*p* < 0.05, Figure 2E) than SC‐P. Cooked GM‐P was darker (L*, *p* < 0.05, Figure 2F), redder (a*, *p* < 0.05, Figure 2G), and less yellow (b*, *p* < 0.05, Figure 2H) than the SC‐P sample.

### 4.3 Starch Digestibility of Bread and Pasta

**FIGURE 3:**
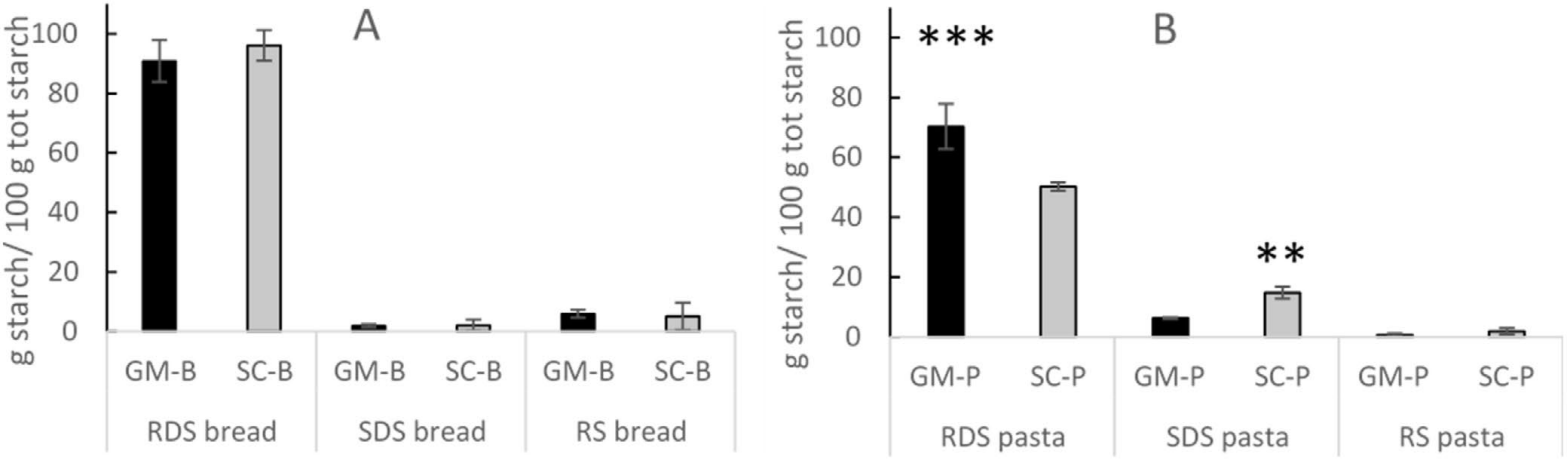
Rapidly digestible starch (RDS), slowly digestible starch (SDS) and resistant starch (RS) of (A) bread and (B) pasta made with Senatore Cappelli (SC‐P; SC‐B) and Grano Mischio (GM‐P; GM‐B) flour. *T*‐test was used to assess the difference between the same product samples made with the different flours: **p < 0.01, ***p < 0.001.

In vitro starch digestibility is shown in Figure 3A,B for bread and pasta, respectively. RDS was the most prominent fraction for bread, accounting for almost the entire starch content (96.0 ± 5.0 g/100 g total starch for SC‐B and 90.0 ± 7.0 g/100 g total starch for GM‐B). In contrast, SDS and RS represented less than 10% of the total starch. No significant differences were observed between bread made with the two flours for any of the starch fractions.

As in bread, RDS of pasta was the dominant starch fraction; however, it accounted for 70.3 ± 7.5 g/100 g total starch in GM‐P, which was significantly (*p* < 0.01) higher than the RDS in SC‐P (50.2 ± 1.4 g/100 g total starch) (Figure 3B). The SDS was significantly (*p* < 0.01) higher in SC‐P (14.8 ± 1.9 g/100 g total starch) compared to GM‐P (6.3 ± 0.3 g/100 g total starch), while no significant differences were observed between the pasta made with the two flours for RS (1.9 ± 1.1 g/100 g total starch for SC‐P and 0.7 ± 0.5 g/100 g total starch for GM‐P).

### 4.4 Antioxidant Activity

**FIGURE 4:**
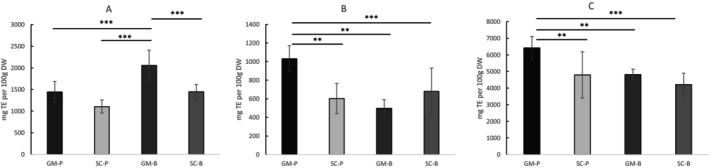
(A) ORAC (B) ABTS, and (C) DPPH assays after in vitro digestion of bread and pasta made with Senatore Cappelli (SC‐P; SC‐B) and Grano Mischio (GM‐P; GM‐B) flour. Data are expressed as mean ± SD per 100 g dry weight (DW). *T*‐test was used to assess the difference between the samples. **p < 0.01, ***p < 0.001.

The antioxidant activity of digested pasta and bread samples was assessed using ORAC, ABTS, and DPPH assays (Figure 4). In the ORAC assay (Figure 4A), GM‐B displayed the highest antioxidant capacity, exceeding not only SC‐B (*p* < 0.001) but also both pastas (vs. SC‐P: *p* < 0.001, vs. GM‐P: *p* < 0.001), therefore indicating that the bread matrix was more effective in preserving ORAC‐measured antioxidant potential for GM. In contrast, the ABTS and DPPH assays (Figure 4B,C) revealed the strongest radical‐scavenging capacity for GM‐P, outperforming SC‐P (*p* < 0.01) and both bread samples (vs. SC‐B: *p* < 0.001; vs. GM‐B: *p* < 0.01). Across all assays, SC products consistently exhibited lower antioxidant activity than their GM equivalents, regardless of the method used.

### 4.5 Cell Viability

**FIGURE 5:**
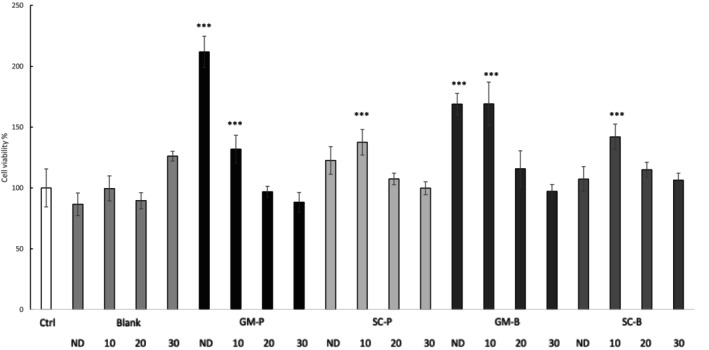
The viability assay (MTT). The cytotoxicity of the digested samples was tested at the following dilutions: ND (non‐diluted), 10 (1:10 v/v), 20 (1:20 v/v), 30 (1:30 v/v). SC‐P; SC‐B: Pasta and bread made with Senatore Cappelli flour, respectively. GM‐P; GM‐B: Pasta and bread made with Grano Mischio flour, respectively. Data are expressed as mean ± SD. *T*‐test was performed between each sample and control group (cells only, ctrl): ***p < 0.001.

Cell viability after exposure to digested samples at various dilutions (non‐diluted, 1:10; 1:20; 1:30 v/v) is presented in Figure 5. None of the samples, at any dilution, exhibited cytotoxicity toward Caco‐2 cells. The highest non‐cytotoxic concentration was recognized as the 1:10 dilution. Additionally, the digestive fluids (blank) did not affect cell viability. The GM samples, both bread and pasta, in their non‐diluted (ND) form and at a 1:10 dilution, significantly (*p* < 0.001) increased cell viability compared to the control (ctrl) group (cells only). Similarly, the SC bread and pasta samples at a 1:10 dilution significantly (*p* < 0.001) enhanced cell viability.

### 4.6 Intestinal Permeability

**FIGURE 6:**
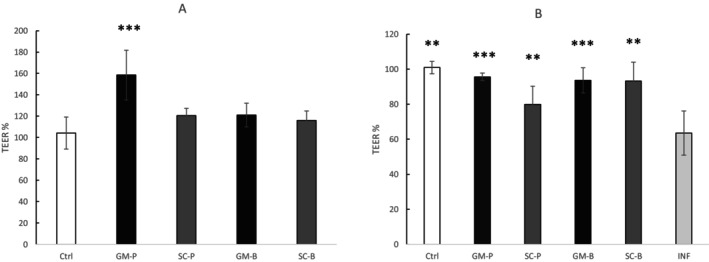
(A) %TEER after 2 h of treatment with the digested samples compared to untreated cells (ctrl). (B) %TEER after 2 h of digesta treatment and 3 h of inflammation with LPS and IL‐1β compared to only the inflamed control (INF). TEER; Transepithelial electrical resistance. SC‐P; SC‐B: Pasta and bread made with Senatore Cappelli flour, respectively. GM‐P; GM‐B: Pasta and bread made with Grano Mischio flour, respectively. Values are presented as the mean ± SD (*n* = 3). ANOVA with Dunnett's post hoc test: Each sample vs. ctrl (Figure 6A), each sample versus INF (Figure 6B). **p < 0.01, ***p < 0.001.

The effect of digesta at the highest non‐cytotoxic concentration (dilution 1:10) on intestinal cell permeability was assessed using TEER, and the results are expressed as the percentage of the initial value (Figure 6). After 2 h of treatment, only cells treated with digested GM‐P exhibited a significant (*p* < 0.001) increase in TEER compared to the initial value, indicating a substantial improvement in epithelial integrity compared to untreated cells (Figure 6A). Following 2 h of digesta treatment and a subsequent 3 h of LPS‐ and IL‐1β‐induced inflammation, all treatments significantly improved epithelial resistance compared to inflamed (INF) and untreated (ctrl) samples, when TEER values were expressed relative to the pre‐inflammation measurements (Figure 6B). In particular, GM‐P and GM‐B displayed the strongest protective effects (*p* < 0.001), restoring TEER values close to baseline, whereas SC‐P and SC‐B showed a milder yet significant protection (*p* < 0.01). Of note, digested GM‐P showed a more pronounced protective effect (+32.06%) compared to digested SC‐P (+16.33%) (Figure 6B).

### 4.7 Modulation of IL‐1B, NF‐kB, and IL‐11 Gene Expression

**FIGURE 7:**
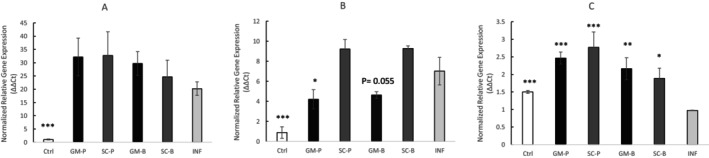
Effects of 3 h inflammatory stimuli on (A) IL‐1B (B) NF‐kB and (C) IL‐11 gene expression levels in a Caco‐2 monolayer epithelium pre‐treated with digested food for 2 h. SC‐P; SC‐B: Pasta and bread made with Senatore Cappelli flour, respectively. GM‐P; GM‐B: Pasta and bread made with Grano Mischio flour, respectively. Values are presented as the mean ± SD (*n* = 3). Results are expressed in relative terms concerning the only inflamed control (INF). *T*‐test sample vs. INF. *p < 0.05, **p < 0.01, ***p < 0.001.

The protective effects of digested GM pasta and bread on inflammation‐induced intestinal epithelium were evaluated by studying the modulation of IL‐1β, NF‐kB, and IL‐11 expression and the results are presented in Figure 7. The expression of IL‐1β and NF‐kB was significantly elevated in the inflamed samples compared to the untreated control (*p* < 0.001, Figure 7A,B), confirming the successful induction of inflammation in the model.

None of the digested products counteracted the inflammation‐induced increase in IL‐1β (Figure 7A). In contrast, NF‐κB expression, a key transcription factor regulating inflammatory responses, was significantly reduced in cells pre‐treated with digested GM‐P (*p* < 0.001). At the same time, GM‐B showed a reduction at the threshold of significance (*p* = 0.055), and no significant modulation was observed for SC‐derived products (Figure 7B). Notably, the reduction in NF‐κB expression was not accompanied by a corresponding decrease in IL‐1β expression. Regarding IL‐11, a cytokine associated with epithelial repair, all digested samples induced significantly higher expression levels than the inflamed control, with the strongest upregulation detected for pasta samples, GM‐P showing the strongest evidence overall (*p* < 0.001, Figure 7C).

### 4.8 Modulation of Tight Junction Gene Expression

Figure S1 (supporting information) shows the effects of a 3 h inflammatory stimulus on gene expression levels of genes encoding for the tight junctions assessed in a Caco‐2 monolayer epithelium pre‐treated with digesta for 2 h, with results expressed in relative terms compared to the inflamed control (INF). The analysis revealed that the expression of ZO‐1 and Claudin was significantly elevated in the inflamed samples compared to the controls (*p* < 0.001 and *p* < 0.01, respectively) (Figure S1B,C). None of the digested samples was able to counterbalance this effect. Also, no significant differences were observed in Occludin gene expression between control groups and any treatments (Figure S1A).

## 5 Discussion

Pigmented wheats have been proposed to confer health benefits due to their richness in polyphenols and resulting antioxidant activity (Dinu et al. 2018; Ed Nignpense et al. 2024; Iannucci et al. 2022). However, few studies have examined how the food matrix, shaped by different processing methods, affects the release and activity of these compounds after digestion. Based on the results of Suo et al. (Suo et al. 2023), who found that pigmented wheat (GM) contains higher levels of antioxidant compounds compared to non‐pigmented wheat (SC), this study investigates the antioxidant activity of digesta from pasta and bread produced from the same flours (GM and SC) but subjected to different processing methods. Additionally, the protective effects of these digesta on intestinal epithelial integrity were evaluated using the Caco‐2 cell line model, with particular attention to inflammatory responses and the modulation of gene expression.

The main findings of this study highlight that both the raw material and the processing method influence the functional properties of these cereal‐based foods. Specifically, the results indicate that the polyphenol‐rich raw material is the primary driver of the observed bioactivity, while the pasta matrix further amplifies this effect compared with bread.

Starting with the food structure, we observed that the use of pigmented wheat significantly influenced the textural properties of both bread and pasta. GM‐B exhibited increased hardness, reduced volume, and lower cohesiveness compared to SC‐B. These results are likely due to the higher polyphenol content of GM wheat, especially ferulic acid, which was approximately 3‐fold higher than that of the traditional SC wheat, as previously quantified by Suo et al. (Suo et al. 2023). Polyphenols, and in particular ferulic acid, interact with gluten by reducing disulfide bonds, which are essential for the formation of the protein network, while increasing thiol groups, thereby diminishing dough elasticity (Nicks et al. 2013; Xu et al. 2019). Furthermore, ferulic acid can form complexes with gluten, reducing the dough's resistance to extension and accelerating its breakdown (Nicks et al. 2013). These phenomena limit expansion during fermentation and baking. In pasta, the use of pigmented wheat slightly increased solid loss to around 5.5%, compared to 4.5% in SC‐P, which aligns with the data reported by Suo et al. (Suo et al. 2023). The presence of polyphenols in GM‐P may have altered the molecular organization of proteins, weakening the protein network and compromising the dense structure of the pasta, consequently increasing solid loss during the cooking process (Krekora et al. 2021; Welc et al. 2022).

The structural features of bread and pasta influence their in vitro starch digestibility. Although GM bread had a lower volume, which is generally associated with reduced starch digestibility due to limited surface area and porosity (Tagliasco, Boukid, et al. 2024), it exhibited a starch digestion level comparable to that of the more porous SC‐B. This may be explained by the lower cohesiveness of GM bread, which increases disintegration during digestion and facilitates starch breakdown (Tagliasco, Font, et al. 2024). A similar trend was observed in pasta, even more markedly. The pasta matrix structure mainly drives digestion; the remarkable presence of polyphenols, which interact with the gluten network, increased the disintegration rate and, consequently, the digestion rate (Gallo et al. 2020). This explains the higher starch digestion observed in GM‐P compared to SC‐P.

Beyond texture, the antioxidant behavior of the products revealed interesting matrix‐dependent patterns. High‐temperature baking, as used for bread production, is known to promote the degradation of thermolabile phenolic compounds, particularly in the crust, where temperatures can exceed 200°C. In contrast, low‐temperature drying (around 50°C), as used for artisanal pasta, together with cooking temperatures that do not exceed 100°C, may help retain a greater fraction of these compounds (Deng et al. 2018). Phenolic acids, such as ferulic and *p*‐coumaric acids, represent the main polyphenols in pigmented wheat and are likely responsible for the majority of the observed antioxidant effects (Cao et al. 2021). These compounds can bind to proteins and polysaccharides within the food matrix, thereby protecting them from degradation during processing and enhancing their bioaccessibility (Deng et al. 2018; Cao et al. 2021).

Interestingly, the antioxidant assays reflected these matrix‐dependent effects. GM‐B exhibited the highest ORAC values, suggesting greater peroxyl radical scavenging capacity, potentially due to the formation or retention of compounds during baking that favor hydrogen atom transfer mechanisms. Conversely, GM‐P showed the highest DPPH and ABTS values, indicating stronger electron‐donating ability, likely attributable to the better retention of thermolabile phenolic acids during low‐temperature drying (Gulcin and Alwasel 2023; Yamauchi et al. 2024). These findings confirm that antioxidant capacity is assay‐dependent, as each test captures distinct chemical reactivities (Apak et al. 2016). Notably, the highest DPPH and ABTS values observed in GM‐P align with its more pronounced protective effect on intestinal barrier function under non‐inflammatory conditions, suggesting a possible role of electron transfer capacity in the observed protective effects. When cells were inflamed with LPS and IL‐1β, all treatments significantly increased TEER relative to inflamed controls, but GM‐P was even more effective than SC‐P. The antioxidant compounds may have counteracted oxidative and inflammatory stress at the epithelial level. Although GM‐P exhibited the highest antioxidant activity and the most pronounced protective effect on intestinal barrier integrity, the underlying mechanisms cannot be conclusively attributed to its antioxidant properties alone. The lack of direct assessment of intracellular oxidative stress (e.g., ROS production, lipid peroxidation) and the absence of pathway‐specific inhibition limit the ability to establish a causal relationship. Therefore, the observed protective effects should be interpreted as potentially, but not exclusively, associated with the antioxidant properties of the samples. Even though GM‐P significantly reduced NF‐κB expression, no corresponding decrease in IL‐1β expression was observed. This may be explained by the fact that the regulation of IL‐1β involves multiple mechanisms beyond NF‐κB activation, including inflammasome‐mediated pathways and post‐transcriptional regulation, as previously reported (Dinarello 2011; Kaszycki and Kim 2025; Lopez‐Castejon and Brough 2011; Guo et al. 2024).

Oxidative stress disrupts tight junction proteins, thereby increasing intestinal permeability (Musch et al. 2006; Chalimeswamy et al. 2022). Antioxidants may mitigate this effect by reducing reactive oxygen species (ROS) levels and preserving the expression and localization of key tight junction proteins (Yang et al. 2017; Martins‐Gomes et al. 2024). Interestingly, in the present study, both ZO‐1 and Claudin expression were increased under inflammatory conditions, reflecting a compensatory cellular response aimed at restoring barrier integrity. This finding may appear counterintuitive, as inflammation is generally associated with tight junction disruption, but it is important to note that mRNA expression levels do not necessarily correlate with protein abundance or localization, which are critical for tight junction functionality. In this experimental setting, no digesta samples significantly affected the expression of tight junction markers, suggesting that the observed effects on barrier integrity are likely mediated through post‐transcriptional mechanisms or the preservation of protein structure and localization rather than transcriptional regulation.

In conclusion, our findings demonstrate that the functional properties of cereal‐based foods arise from the interplay between raw material composition and the structural features developed during processing. Pigmented wheat (GM), through its higher polyphenol content compared to the traditional SC, was the main driver of the observed biological responses, confirming that the polyphenol‐rich ingredient is a primary driver of the observed bioactivity. However, the food matrix significantly modulated these effects (Aguilera 2025). Despite being produced from the same raw materials, pasta and bread showed different functional outcomes after digestion. In particular, digested GM pasta exhibited higher radical‐scavenging capacity in the DPPH and ABTS assays than the other analyzed samples and showed the strongest biological effects on intestinal epithelial cells, including increased cell viability, improved epithelial barrier integrity, and reduced NF‐κB expression under inflammatory conditions. These findings suggest that the dense structure of the pasta matrix may favor the retention and delivery of bioactive compounds during digestion, enhancing their biological efficacy compared with bread. To further elucidate the relationship between the food matrix and antioxidant activity, and to strengthen the overall findings, future studies could incorporate quantitative analyses of the gluten network at the microscopic level, for example through imaging techniques (e.g., confocal laser scanning microscopy) or chemical methods (e.g., disulfide bond quantification).

Overall, the results highlight the importance of considering both ingredient composition and food structure when designing cereal‐based foods with improved functional properties. In this context, pasta produced from pigmented wheat may represent a promising strategy to deliver antioxidant compounds and support intestinal epithelial protection.

## Author Contributions

**Giulia Feliziani:** methodology, data curation, investigation, formal analysis, writing – original draft, conceptualization, visualization. **Laura Bordoni:** conceptualization, validation, supervision, writing – review and editing. **Elena Vittadini:** conceptualization, validation, supervision, funding acquisition, writing – review and editing. **Rosita Gabbianelli:** conceptualization, validation, writing – review and editing, funding acquisition, supervision. **Marianna Tagliasco:** conceptualization, methodology, data curation, investigation, formal analysis, writing – original draft, visualization. **Xinying Suo:** investigation, formal analysis. **Nicoletta Pellegrini:** conceptualization, supervision, funding acquisition, writing – review and editing, validation.

## Funding

The authors have nothing to report.

## Conflicts of Interest

The authors declare no conflicts of interest.

## Supporting information


**Table S1:** Primer sequences for housekeeping genes and genes of interest for qPCR.
**Figure S1:** Effects of digested food pre‐treatment on Occludin (A) Zonula Occludens‐1 (B) and Claudin (C) gene expression in Caco‐2 cells following a 3‐h inflammatory stimulus with LPS and IL‐1B.

## Data Availability

The data that support the findings of this study are available from the corresponding author upon reasonable request.
