# Supplementary material for: Food Structure Modulation of Antioxidant Bioaccessibility and Gut Epithelial Protection: The Case of Bread and Pasta Made With Pigmented Wheat
Source: Food Sci Nutr. 2026 May 3;14(5):e71863. doi: 10.1002/fsn3.71863 (PMC13136512; doi:10.1002/fsn3.71863)
Supplement: Supplementary file 1 — Table S1: Primer sequences for housekeeping genes and genes of interest for qPCR. Figure S1: Effects of digested food pre‐treatment on Occludin (A) Zonula Occludens‐1 (B) and Claudin (C) gene expression in Caco‐2 cells following a 3‐h inflammatory stimulus with LPS and IL‐1B. [file FSN3-14-e71863-s001.docx]

**Supplementary Figure S1. Effects of digested food pre-treatment on Occludin (A), Zonula Occludens-1 (B) and Claudin (C) gene expression in Caco-2 cells following a 3-hour inflammatory stimulus with LPS and IL-1B.**


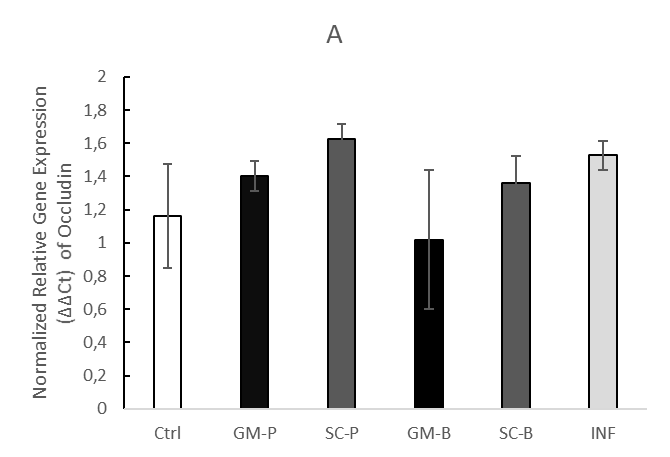

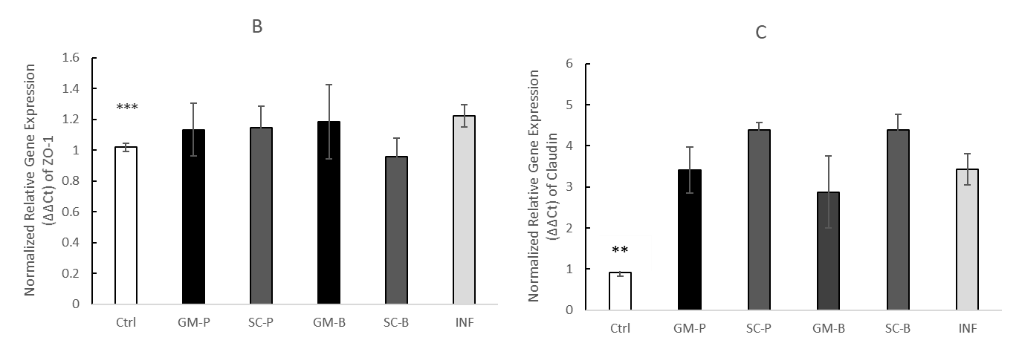


SC-P; SC-B: pasta and bread made with Senatore Cappelli flour, GM-P; GM-B: pasta and bread made with Grano Mischio flour. Values are presented as the mean ± SD (n=3). Results are expressed in relative terms concerning the only inflamed control (INF). t-test sample vs INF. * p<0.05, ** p<0.01, *** p<0.001.

**Supplementary Table** **S1:** **Primer sequences for housekeeping genes and genes of interest for qPCR**

| **GENE** | **NAME** | **FW** | **RV** | **MELTING T** |
| --- | --- | --- | --- | --- |
| **IL-11** | Interleukin 11 | GGA CAG GGA AGG GTT AAA GG | GCT CAG CAC GAC CAG GAC | 60/61 |
| **IL-1β** | Interleukin 1β | AGA TGA TAA GCC CAC TCT ACA G | ACA TTC AGC ACA GGA CTC TC | 60/58 |
| **NF-kB** | Nuclear factor kappa-light-chain-enhancer of activated B cells | ACA GCT GGA TGT GTG ACT GG | TCC TCC GAA GCT GGA CAA AC | 60 |
| **ZO-1** | Zonula Occludens-1 | TTC ACG CAG TTA CGA GCA AG | TTG GTG TTT GAA GGC AGA GC | 58 |
| **OCCLUDIN** | Occludin | GGG CAT TGC TCA TCC TGA AG | GCC TGT AAG GAG GTG GAC TT | 60 |
| **CLAUDIN-1** | Claudin-1 | TGG TCA GGC TCT CTT CAC TG | TTG GAT AGG GCC TTG GTG TT | 60/58 |
| **BACT** | Β-Actina | GAA GAT CAA GAT CAT TGC TC | ATC CAC ATC TGC TGG AAG G | 61/57 |
